# Positive real-time PCR in pneumococcal meningitis 12 hours after initiation of antibiotic therapy – case report

**DOI:** 10.1186/s12883-025-04033-7

**Published:** 2025-01-22

**Authors:** Cem Thunstedt, Carla Palleis, Johannes Wischmann, Suzette Heck, Konstantinos Dimitriadis, Matthias Klein

**Affiliations:** 1 Department of Neurology, LMU University Hospital, LMU Munich, Marchioninistrasse 15, 81377 Munich, Germany; 2 Emergency Department, LMU University Hospital, LMU Munich, Munich, Germany

**Keywords:** Meningitis, Pneumococcal, Polymerase chain reaction, Multiplex

## Abstract

**Background:**

Purulent meningitis poses a significant clinical challenge with high mortality. We present the case of a 54-year-old female transferred to our emergency department with suspected bacterial meningitis, later diagnosed as an Austrian syndrome.

**Case presentation:**

The patient exhibited subacute somnolence, severe headache, nausea and fever. Initial antibiotic therapy was initiated without successful lumbar puncture. Upon arrival at our hospital, she presented with septic shock, meningism, and respiratory symptoms. Lumbar puncture revealed cloudy cerebrospinal fluid with elevated cell count, protein, and low glucose. While blood and CSF cultures remained negative, multiplex PCR for *Streptococcus pneumoniae* was positive even 10 h after beginning of effective antibiotic therapy. Subsequent echocardiogram revealed mitral valve endocarditis and the patient underwent valve replacement.

**Conclusion:**

Altogether, bacterial meningitis presents with cardinal clinical signs only half of cases. Lumbar puncture remains crucial, and our patient’s CSF findings aligned with bacterial meningitis. Multiplex PCR aided in diagnosis, even after antibiotic treatment. The case highlights the importance of prompt lumbar puncture despite antibiotic pre-treatment. The patient’s Austrian syndrome, characterized by meningitis, endocarditis, and pneumonia, emphasizes the need for vigilance regarding skin lesions, early cerebral infarctions, and iritis. This case emphasizes the complexity of bacterial meningitis diagnosis and the utility of multiplex PCR, especially in prolonged antibiotic-treated patients. However, PCR cannot replace cultures when it comes to adapting therapy based on the antibiotic sensitivity of the causative pathogen. Awareness of Austrian syndrome’s diverse manifestations is crucial for timely recognition and appropriate management.

## Background

Bacterial meningitis still poses a clinical challenge, as it is associated with a mortality rate of up to 21% [1]. Furthermore, diagnosis is complicated by the fact that classical symptoms such as headache, meningism, altered consciousness, and fever are observed in only approximately half of all cases, as exemplified by our patient [1]. To diagnose acute bacterial meningitis, lumbar puncture is crucial, which however is sometimes anatomically not possible or contraindicated. Therefore, we present a case, in which lumbar puncture was initially unsuccessful.

## Case

**Fig. 1:**
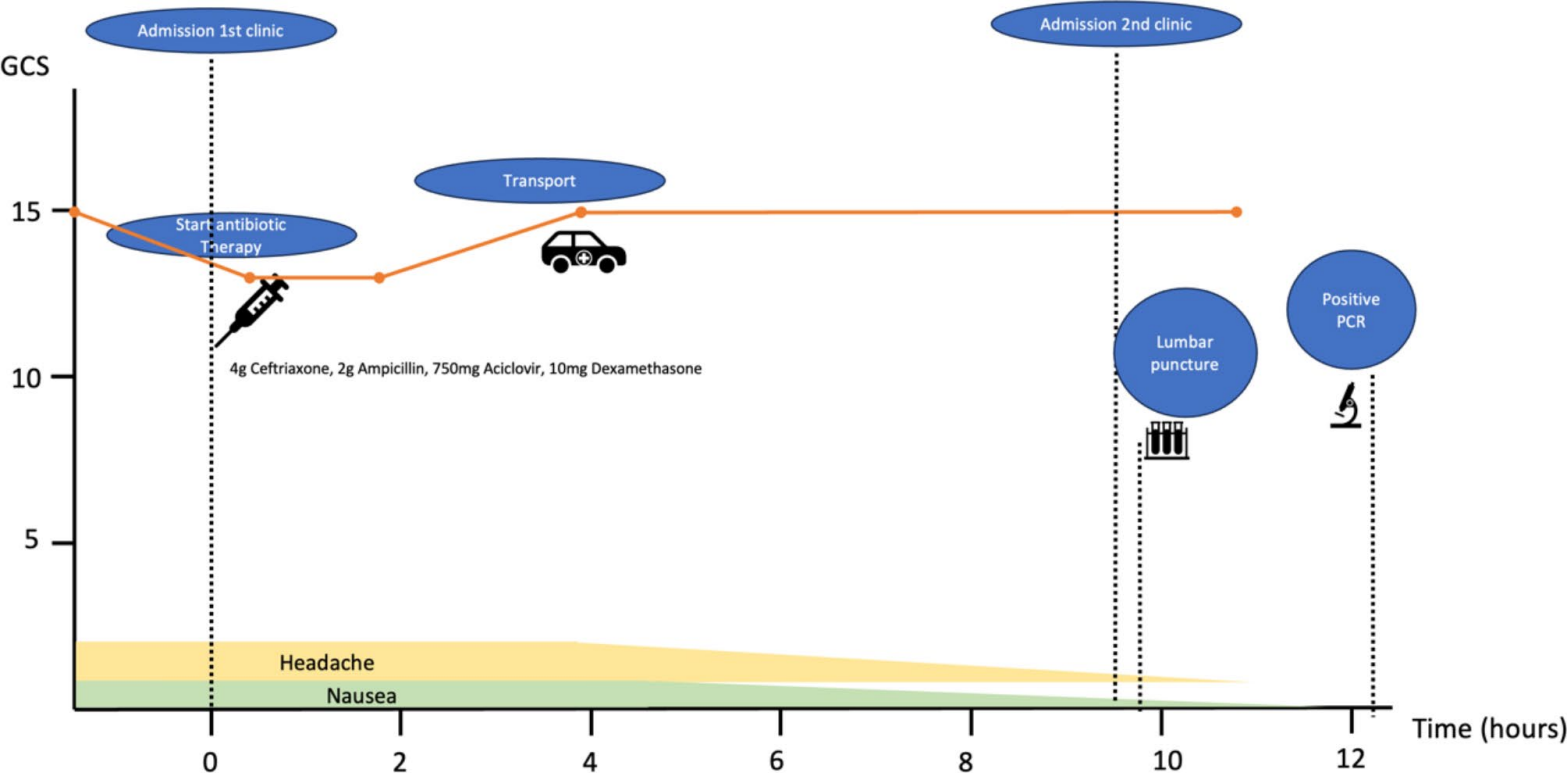
Timeline

**Fig. 2:**
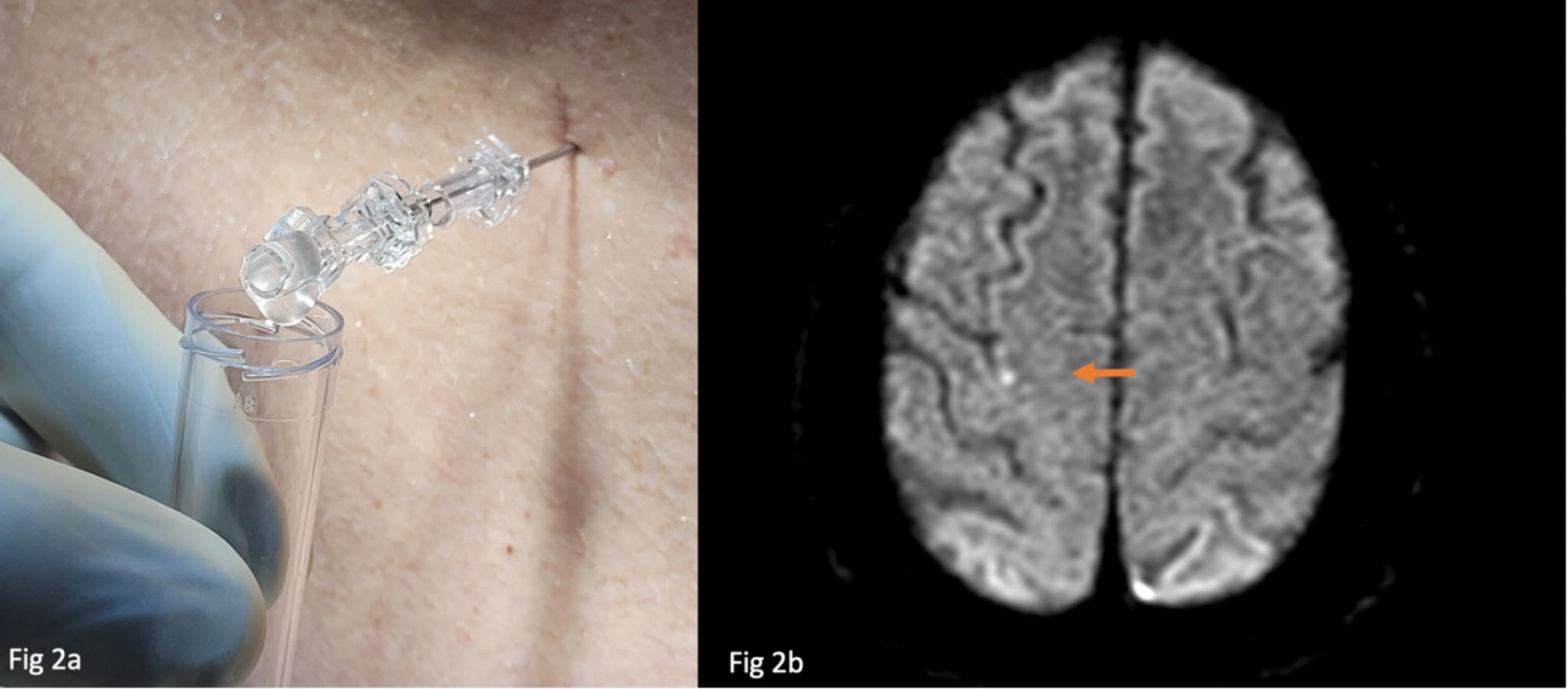
**2a** Macroscopic CSF fluid: turbid CSF detected at lumbar puncture indicating purulent meningitis. **2b**: Axial MRI-DWI of the brain revealing diffusion restriction (arrow) indicating embolic stroke, highly suggestive of Austrian syndrome in the patient with pneumococcal meningitis and pneumonia

A 54-year-old female patient was transferred to our emergency department from an external hospital because of suspected bacterial meningitis. Initial presentation at the external hospital included subacute somnolence (Glasgow Coma Scale [2] (GCS) of 13), bitemporal headache, nausea/vomiting and fever (40 °C). The patient received intravenous antibiotic therapy according to guideline recommendations, consisting of 4 g ceftriaxone, 2 g ampicillin, 750 mg aciclovir and 10 mg dexamethasone after blood cultures were obtained. Computed tomography (CT) scan revealed no abnormal findings. Lumbar puncture was attempted but was unsuccessful as only a few cloudy drops were seen in the conus of the puncture needle and no CSF could be collected for further evaluation. On arrival at our hospital (about 10 h later (Fig. 1)), the patient remained somnolent (GCS 13), displayed meningism, and was diagnosed with septic shock requiring vasopressors (systolic blood pressure of 100 mmHg with norepinephrine 0.8 mg/h, heart rate > 100/min, serum lactate 13 mmol/l and procalcitonin 3 µg/l). Additional blood cultures were taken and the above antibiotic regimen was continued. A lumbar puncture was performed, revealing cloudy and turbid CSF fluid upon visual examination (Fig. 2a); 3064 cells/µl, 174 protein mg/dl and a low glucose of 35 mg/dl (glucose Index 0.22) (Table 1). Serum lactate levels improved (from 3 to 1mmol/l) after fluid administration. The patient’s level of consciousness also improved (GCS 15). Respiratory symptoms were noted, prompting an immediate chest CT-pulmonary angiogram, which excluded pulmonary artery embolism, but revealed bilateral infiltrates and pleural effusion indicating community-acquired pneumonia. The patient was then transferred to our neurological intensive care unit. During the treatment process, the pathogen diagnostics via the multiplex-(polymerase chain reaction) PCR *(BioFire FilmArray* ^®^*)* showed a positive result for *S. pneumoniae* in the CSF taken 12 h after the initiation of antibiotic administration, while gram stain, single-PCR (*BD MAX™ System assay for ply und lytA*) and all culture (CSF and blood by first and second clinic) remained negative. Also, all blood cultures were found negative. Subsequent MRI of the brain revealed extra-axial small punctual restricted diffusion in terms of septic embolism without hydrocephalus, vein-thrombosis, empyema, cerebritis or ventriculitis (Fig. 2b). Furthermore, ophthalmologic examination revealed iritis. Ceftriaxone and ampicillin were administered for 7 days, while aciclovir was discontinued after 2 days following a negative herpes simplex PCR result. A transesophageal echocardiogram subsequently confirmed native mitral valve endocarditis. Ampicillin therapy was continued for an additional 3 weeks, with gentamicin added for a 2-week overlap, in accordance with the ESC guidelines for infective endocarditis valid at the time [3].

**Table 1:** Lumbar puncture, laboratory and microbiology results (abnormal results: bold)

CSF	Value
Cell count (cells/µl)	**3046**
Protein (mg/dl)	**174**
Glucose (mg/dl)	**35**
Serum-CSF Glucose Index	**0**,**22**
**Blood**	
Glucose (mg/dl)	**155**
CRP (mg/dl)	**25**,**3**
PCT (ng/ml)	**6**,**3**
Leukocytes (G/l)	**22**,**9**
Lactate (mmol/l)	1
Culture	-
**Mikrobiology/virology results for** ***Streptococcus pneumoniae***	
Multiplex PCR	**+**
Single PCR	-
Gram staining	-
CSF-culture (cumulative)	-

The combination of pneumococcal meningitis with concomitant pneumonia and endocarditis enabled the final diagnosis of an Austrian syndrome. Due to acute cardiac decompensation caused by mitral valve insufficiency with anterior mitral leaflet perforation, valve replacement had to be performed acutely despite the risk of cerebral hemorrhage. The patient recovered well after surgical valve replacement and showed no long-term neurological sequelae (3-month follow-up).

## Discussion and conclusions

In this case, the patient finally was found to suffer from an Austrian syndrome, a rare but life-threatening condition characterized by the simultaneous presence of pneumococcal meningitis endocarditis and pneumonia, most likely as septic embolic spread from pneumonia, with subsequent cardiac and CNS-involvement, all of which our patient suffered from. Death rate ranges from 60 − 32% due to sepsis, septic shock or cardiac failure. It mainly affects immunocompromised patients or patients with alcohol abuse, which our patient did not fulfil. Red flags that should prompt the treating physician at this diagnosis in the circumstance of pneumococcal meningitis are the presence of fever, new cardiac murmur, skin lesions (Osler spots) and/or early cerebral infarctions. Another sign that can often be found is the presence of ocular manifestation (i.e. iritis), which was also detected in our patient [4]. The Austrian syndrome was named according to the publication of a case by the physician Robert Austrian in 1957 [5]. Similar syndromes had previously been described, however, by Richard Heschl in the 1860s and by Wiliam Osler. However, both had not established a link with an infection by Streptococcus pneumoniae [6]. This pathogen is a rare cause of endocarditis, particularly native-valve endocarditis, which is more commonly observed in immunocompromised patients and is associated with high mortality. While typically limited to case reports, the incidence is notably higher in Alaska and Denmark. Infectious native-valve endocarditis associated with Austrian syndrome has been documented but remains an exceptionally rare condition [7]. 

Considering the diagnostic regimen, our case revealed turbid and cloudy CSF, with evidence of granulocytic pleocytosis (> 1000 cells/µl), elevated protein levels, and hypoglycorrhachia [8]. These findings allow the diagnosis of bacterial meningitis. Our patients additionally showed elevated serum procalcitonin levels, which have also been identified as a valuable indicator of bacterial meningitis, distinguishing it from aseptic meningitis with sensitivity and specificity rates of 99 and 80%, respectively [9]. For microbiologic workup, we used multiplex PCR testing which revealed *S. pneumoniae.* This is remarkable because all other test results (including blood culture, CSF culture and gram stain from CSF) showed negative results. However, the negativity of the culture and gram stain that were ordered from samples taken in the emergency department of our hospital can well be explained as the patient had been treated with adequate antibiotics for a total of 12 h already. One explanation why PCR was still positive in this situation could be that it allowed detection of genetic material from already killed bacteria, at a time point when viable bacteria could not be found any more. The finding that PCR can still be positive in patients where all other conventional tests fail to detect the pathogen (especially when samples are drawn after antibiotics were applied) is in line with single previous reports in the literature [10]. Positive PCR results after starting antibiotic therapy for bacterial meningitis were seen as late as 48 h after cefuroxime was begun in single patients [10, 11]. Due to the limited data, however, it remains unclear what the cut-off in terms of time after the start of antibiotic therapy is for PCR and how quickly sensitivity of PCR (and the other diagnostic methods) decrease in meningitis after treatment with antibiotics is begun. All in all, however, our case report and the reports published previously strongly support the use of (multiplex) PCR techniques, especially in patients who receive antibiotics before CSF is obtained. In this context, however, it is of great importance to point out that the possibilities of PCR testing should not overshadow the necessity of seeking for an early lumbar puncture [12–14]. Furthermore, PCR cannot replace blood and CSF cultures as they are currently the only method that allows to obtain the antibiotic sensitivity of the causative pathogen. An integration of common resistance sequences in the PCR techniques used would be desirable.

Concerning the therapeutic regimen for Austrian syndrome, optimal antibiotic therapy remains unclear, as no randomized studies are available. Treatment with a cephalosporin is usually undertaken. Here, we opted for ampicillin/gentamicin based on current ESC guidelines for streptococcal endocarditis, given the relatively low resistance rate to penicillin in Germany (approximately 5%) and the patient improved. However, the use of empiric penicillin in absence of antibiotic susceptibility testing of the isolated bacteria needs to be considered carefully, especially as clear cut-off points for acceptable resistance rates to do this are missing. In cases of doubt, treatment with a cephalosporin is advisable. The optimal duration of therapy is likewise unclear; a case series shows large differences in treatment duration ranging from 2 to 12 weeks. However, based on the patients rapid improvement, we agreed to limit the treatment duration to 4 weeks as recommended for streptococcal endocarditis [3, 15, 16].

In conclusion, when community-acquired bacterial meningitis is suspected, the main objective is to establish a diagnosis and promptly initiate empirical therapy. Evaluation of the CSF is key. In patients pre-treated with antibiotics, the use of (multiplex) PCR techniques in addition to gram stain and culture seems to increase the diagnostic yield.

## Data Availability

No datasets were generated or analysed during the current study.

## Abbreviations

CSF: Cerebrospinal fluid
CT: Computed tomography
PCR: Polymerase chain reaction
